# pH-Sensitive Naproxen Delivery via ZIF and Kaolin@ZIF Nanocarriers in 3D-Printed PLA–Gelatin Hydrogels

**DOI:** 10.3390/polym17182497

**Published:** 2025-09-16

**Authors:** Reyhan Çetin, Berna Ates, Ozgul Gok, Birgül Benli

**Affiliations:** 1 Nanoscience and Nanoengineering Graduate Program, İstanbul Technical University, 34469 Istanbul, Turkey; cetinr21@itu.edu.tr; 2 Department of Biomedical Engineering, Graduate School of Natural and Applied Sciences, Acıbadem Mehmet Ali Aydınlar University, 34752 Istanbul, Turkey; berna.ates@live.acibadem.edu.tr; 3 Department of Biomedical Engineering, Faculty of Engineering and Natural Sciences, Acıbadem Mehmet Ali Aydınlar University, 34752 Istanbul, Turkey; 4 Department of Mineral Processing Engineering, İstanbul Technical University, 34469 Istanbul, Turkey

**Keywords:** pH-responsive drug delivery, zeolitic imidazolate framework (ZIF), kaolin, nanocarrier, 3D printing, polylactic acid (PLA), gelatin hydrogel, naproxen

## Abstract

This study presents a pH-responsive drug delivery platform, created based on naproxen-loaded zeolitic imidazolate frameworks (ZIF) and kaolin-ZIF (Kao@ZIF) nanocarriers embedded in a 3D-printed polylactic acid (PLA) scaffold coated with a gelatin hydrogel. The PLA discs were designed as structural tissue models to simulate localized drug release. Kaolin (Kao), a basic mineral in the kaolin group that includes halloysite, was selected as a chemically stable and biocompatible adsorbent to enhance ZIF integrity and system reliability. To address the concerns about the safety and reproducibility of nanoscale materials in biomedical applications, structurally stable ZIF and Kao@ZIF nanocarriers were synthesized and characterized using FT-IR, SEM-EDS, and LC-M/MS, measuring drug loading efficiencies over 90% for ZIF and slightly higher for Kao@ZIF. In vitro release profiles showed strong pH sensitivity, with greater naproxen release at acidic pH (5.4) and more sustained release from Kao@ZIF. Cytotoxicity assays using L929 fibroblasts demonstrated improved biocompatibility, with cell viabilities of approximately 75% for ZIF–naproxen, 82% for Kao@ZIF–naproxen, and 90% for gelatin-coated PLA–Kao@ZIF scaffolds, for 24 h incubation. Incorporating kaolin-stabilized ZIF nanocarriers into 3D-printed biodegradable scaffolds offers a promising and safer approach for pH-sensitive, tissue-targeted drug delivery, while laying the groundwork for future studies involving halloysite-derived nanotubular systems.

## 1. Introduction

Advancements in nanotechnology have significantly transformed the field of drug delivery by enabling precise, targeted, and controlled therapeutic applications. Among these innovations, nanocarriers—nanoscale systems designed to encapsulate and transport drugs—have emerged as a key technology which improve solubility, bioavailability, and site-specific release [1,2]. These systems are broadly categorized into organic (e.g., liposomes, micelles, polymeric nanoparticles) and inorganic (e.g., metallic, ceramic, or hybrid materials) systems. While organic carriers offer biocompatibility and flexibility, they often suffer from structural instability and limited drug loading. Conversely, inorganic nanocarriers, particularly metal–organic frameworks (MOFs), provide structural integrity, large surface areas, and tunable porosity, making them ideal for stimuli-responsive drug delivery [3,4]. A particularly promising subclass of MOFs is the Zeolitic Imidazolate Frameworks (ZIFs), which combine the thermal and chemical stability of zeolites with the structural flexibility of organic linkers. ZIFs have gained attention for their high drug loading capacity, pH-responsive release profiles, and enhanced biocompatibility [5,6]. However, challenges such as limited colloidal stability, burst release, and clinical translation hinder their stand-alone use. To overcome these issues, hybrid systems that incorporate ZIFs with pharmaceutically crucial materials are being explored to overcome these issues.

In this study, kaolin (Kao@)—a naturally abundant, pharmacopeia-approved clay composed of hydrated aluminum silicate (Al_4_Si_4_O_10_(OH)_8_), with a theoretical chemical composition of 46.54% SiO_2_, 39.50% Al_2_O_3_, and 13.96% H_2_O—was selected as a support material to enhance the structural integrity, regulatory compliance, and biocompatibility of ZIF-based nanocarriers [7]. Unlike halloysite nanotubes (HNTs), kaolin presents a non-tubular layered morphology, contributing to improved mechanical strength, thermal stability, and drug retention, while still allowing surface functionalization [8]. Although kaolin has long been used as a pharmaceutical excipient (e.g., flow regulator or filler) and is recognized by both USP and Ph. Eur. Regulations [9], its application as advanced nanocarrier systems (in cosmetics and wound healing materials, and integration with ZIFs in drug delivery platforms) remains largely unexplored. Traditionally considered an inert additive, kaolin’s high surface area, chemical stability, and favorable surface interactions make it an ideal substrate for anchoring ZIF crystals. Binding ZIFs onto kaolin surfaces (forming Kao@ZIF hybrids) offers several benefits, including enhanced colloidal stability, mitigated burst release, and preservation of ZIF’s functional properties—while maintaining compliance with pharmaceutical safety regulations.

To investigate this hybrid system, we synthesized and characterized both ZIF and Kao@ZIF nanocarriers, which were subsequently loaded with Naproxen, a model nonsteroidal anti-inflammatory drug (NSAID), to obtain a hybrid system. These nanocarriers were then embedded into a gelatin hydrogel and poly (lactic acid) (PLA) scaffold composite, forming a pH-responsive bio-composite patch with potential applications in topical and wound-targeted drug delivery. While ZIFs contribute high drug loading efficiency and better pH-responsive release behavior, the kaolin enhances mechanical stability and biocompatibility, offering a synergistic approach to controlled therapeutic delivery approach.

By merging the performance advantages of ZIFs with the regulatory reliability and safety profile of kaolin, this study introduces a clinically relevant hybrid nanocarrier design that bridges the gap between innovative materials and translational medicine. Accordingly, we investigated the physicochemical and biological performance of the Kao@ZIF system, focusing on drug loading, release kinetics, and cytocompatibility.

Simultaneously, PLA was selected as a scaffold material for its mechanical durability and biodegradability as a scaffold material, and gelatin hydrogel for its excellent biocompatibility and capacity to mimic the natural extracellular matrix. This PLA–gelatin-matrix, reinforced with either ZIF or Kao@ZIF nanocarriers, was fabricated via 3D printing to generate drug-loaded patches tailored for localized, sustained release applications.

Naproxen was chosen for its clinical relevance and pH-sensitive solubility, which makes it an ideal candidate for evaluating stimuli-responsive delivery platforms. As a weakly acidic molecule, its solubility varies significantly across physiologically relevant pH levels, making it well-suited for mimicking drug behavior in environments such as the gastrointestinal tract or wound sites.

Overall, this study presents a novel approach that leverages the complementary properties of ZIFs and kaolin, embedded within a hydrogel–PLA matrix, to create an effective, biocompatible, and regulation-facilitating drug delivery platform suitable for NSAID administration and potentially adaptable to broader therapeutic applications.

## 2. Materials and Methods

### 2.1. Materials

Polylactic Acid (PLA) filament (1.75 mm, matte black, eSUN, Shenzhen, China), pure gelatin powder (food quality) and glutaraldehyde solution (25% *w*/*w* in water; GA, Santa Cruz Biotechnology, Dallas, TX, USA), ethanol (EtOH, ≥70%, Sigma-Aldrich, St. Louis, MO, USA), Kaolin (Kao, powder, Santa Cruz Biotechnology, Dallas, TX, USA), zinc acetate dihydrate (Sigma-Aldrich, St. Louis, MO, USA), 2-methylimidazole (2-mIM, >99%, Isolab, Eschau, Germany) was used. Naproxen, as the model drug, was obtained from Santa Cruz Biotechnology. For nanocarrier characterization and drug loading/release analysis, phosphate-buffered saline (PBS, pH 7.4, Bioshop, Burlington, ON, Canada) and acetate buffer solution (pH 5.2, Sigma-Aldrich, St. Louis, MO, USA) were employed. Cell Counting Kit-8 (CCK-8) was used to assess cell viability and toxicity against L929 fibroblast cells.

### 2.2. Design and Fabrication of the PLA Scaffold

**Figure 1:**
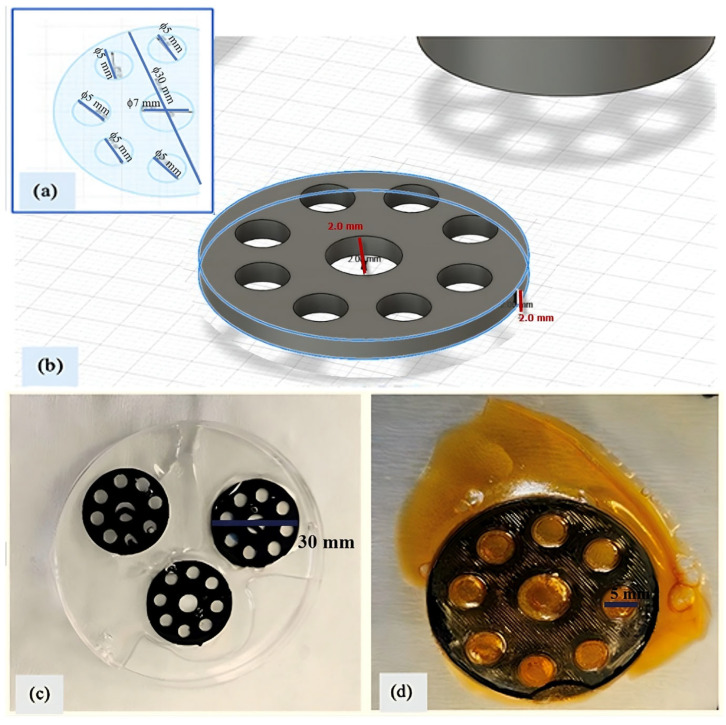
PLA scaffold: (**a**) disc model with inner pores; (**b**) 3D design with 2 mm height; (**c**) gelatin (10%)-embedded scaffold; (**d**) freeze-dried final scaffold.

A circular model with one central hole and seven surrounding pore holes was designed for fabricating the PLA scaffold using Autodesk Fusion 360, Autodesk Inc., San Rafael, CA, USA. The diameter of the disc was 30 mm, with the surrounding pores being 5 mm in diameter and the central hole 7 mm, as shown in Figure 1a. The height of the scaffold was set to 2 mm (Figure 1b). Scaffolds of this low height provide temporary structural support while being easily removable and minimizing material consumption. In biomedical applications, this design enhances cell attachment and tissue integration, improves biocompatibility while interacting with the host environment, and facilitates controlled drug release by promoting the efficient diffusion of bioactive agents. Additionally, it offers practical advantages including improved print quality, ease of handling, aesthetic appeal, and post-processing convenience, making it a preferred configuration.

The PLA scaffold designed in this study was inspired by 3D-printed PLA-based scaffolds coated with hydrogel layers as reported by Schneider et al. [10], aiming to support the biological performance of the final construct in nanomedicine and drug delivery applications. The model shown in Figure 1a was exported as an STL file using Autodesk Fusion 360. The STL file was arranged horizontally in Ultimaker Cura 5.0.0 (Ultimaker B.V., Utrecht, The Netherlands) to maximize yield on the build plate. G-code was generated with the following settings: 20% infill density, build plate adhesion enabled, and no support structure. Additional 3D printer settings were configured as follows: nozzle temperature 200 °C, bed temperature 60 °C, and print speed 50 mm/s. PLA scaffolds were printed horizontally using a Creality Ender-3 V2 (Shenzhen Creality 3D Technology Co., Shenzhen, China) fused deposition modeling (FDM) printer.

### 2.3. Preparation of PLA Scaffolds Embedded with Gelatin and Modified with Glutaraldehyde

The dry weights of the PLA scaffolds (W_PLA_) were recorded before the coating process. The scaffolds were then placed in Petri dishes, and a 10% (*w*/*v*) gelatin solution was applied to all surfaces. Approximately 25 mL of gelatin solution was coated onto each scaffold (Figure 1c). The scaffolds were left to dry overnight in a hood, resulting in a total of 12 layers of gelatin-coated PLA scaffolds. After drying, the scaffolds were weighed again to determine the gelatin uptake (W_PLA&Gelatin_).

Glutaraldehyde was employed as a crosslinking agent due to its widespread use in tissue and bioengineering to enhance the mechanical stability of water-soluble biopolymers like gelatin. It reduces gelatin solubility, increases water resistance, improves durability, and may confer antimicrobial properties. In this process, a 50% (*v*/*v*) glutaraldehyde solution was prepared in 60 mL of distilled water and distributed in 5 mL portions into Petri dishes containing the dried gelatin-coated scaffolds. The Petri dishes were placed in a hood overnight to allow crosslinking of the gelatin polymer chains. Following this, the scaffolds were rinsed with distilled water to remove excess reagent. The samples were then frozen at −80 °C overnight and subsequently lyophilized at the same temperature (Figure 1d). Final weights were recorded to calculate the hydrogel mass formed on scaffolds. The weight of gelatin hydrogel (W_gel_) was determined following the method described by Motakef-Kazemi et al. [11], using Equation (1), where the dry weight of the PLA scaffold (WPLA) was considered negligible and thus excluded from further calculations:

(1) $$W_{hydrogel}\left(mg\right)=W_{PLA\&Gelatin}-W_{PLA}$$

### 2.4. Synthesis of ZIF Nanocarriers

Typically, water-based synthesis methods have been used to prepare ZIF nanocrystals at room temperature [12,13], and were followed in this study as described: 1968 mg of 2-methylimidazole (2-mIM) and 2634 mg of zinc acetate were separately dissolved in 150 mL of distilled water, with stirring at 400 rpm for 2 h at room temperature (~23 ± 2 °C). The zinc acetate solution was then added to the 2-mIM solution. After aging for 2 h, the resulting precipitate was transferred to 50 mL falcon tubes and separated via centrifugation at 4000 rpm for 20 min. The obtained ZIF precipitates were washed three times with distilled water and then transferred to the Petri dishes. To facilitate drying, the Petri dishes were covered with perforated Parafilm and left overnight in a fume hood. The dried samples were then used for further characterization.

### 2.5. Synthesis of Kao@ZIF Nanocarrier Composites

Kaolin (0.35 g) was dispersed in 10 mL of EtOH, and 1653 mg of 2-mIM was dissolved in 40 mL of EtOH and stirred with ultrasound for 30 min in the hood. This solution was then mixed with 0.439 g of zinc acetate dihydrate dissolved in 10 mL of EtOH, following a modified method based on Hou and Wu [14]. After an additional 30 min of sonification to ensure complete dispersion, the composite solution was transferred to 50 mL falcon tubes and centrifuged at 4000 rpm for 20 min. The resulting white precipitate, corresponding to Kao@ZIF nanocomposites, was washed three times with EtOH and several times with distilled water. The final product was placed in a Petri dish, covered with perforated parafilm, and dried overnight in a hood. The dried Kao@ZIF nanocarriers were used for subsequent analysis, and SEM images were obtained for both ZIF and Kao@ZIF samples.

### 2.6. Fabrication of Naproxen-Loaded ZIF and Kao@ZIF Composite for PLA-Embedded Glutaraldehyde-Modified Gelatin Hydrogel

The fabrication of Naproxen-loaded ZIF and Kao@ZIF composites began with the preparation of a 10% (*w*/*w*) Naproxen stock solution, in which 1 mg of Naproxen was dissolved in 200 µL of absolute EtOH using a vortex mixer. For drug loading, 10 mg of synthesized ZIF was dispersed in 5 mL of EtOH and stirred magnetically for 1 h. Then, 1 mL of the prepared Naproxen solution was added to the ZIF dispersion, and the mixture was stirred at 200 rpm for 2 h.

The resulting suspension was transferred into 50 mL falcon tubes and centrifuged at 4000 rpm for 20 min. After phase separation, the supernatant was collected using a micropipette to determine the concentration of unbound Naproxen. The precipitate, referred to as Nap-ZIF, was washed with distilled water and dried under ambient conditions in a petri dish.

**Figure 2:**
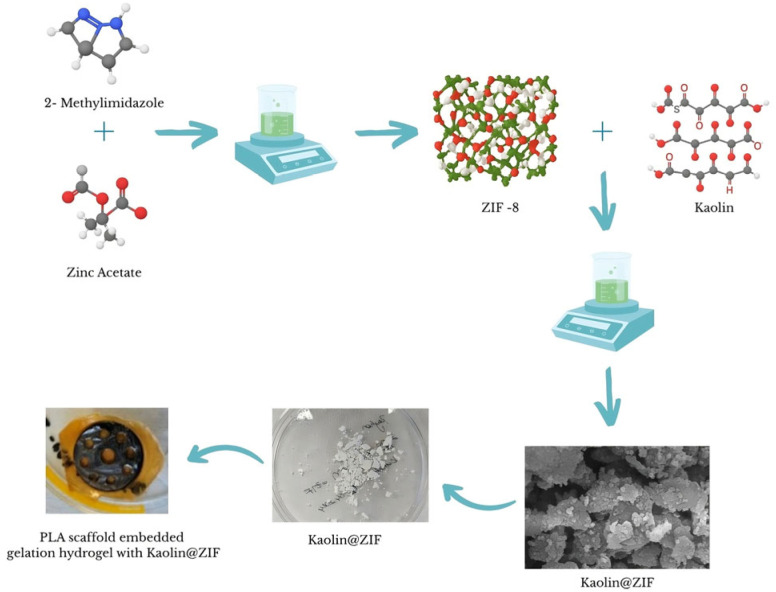
Schematic representation of the synthesis and Naproxen loading process for ZIF and Kao@ZIF nanocarriers embedded in the PLA scaffold.

A similar procedure was followed for the Kao@ZIF composite: 10 mg of Kao@ZIF was suspended in 5 mL of absolute EtOH and stirred magnetically for 1 h. Then, 1 mg of the Naproxen solution was added, followed by stirring at 200 rpm for 2 h. After mixing, the solution was centrifuged under the same conditions. The supernatant was collected for the analysis of drug loading efficiency, and the precipitate, referred to as Nap-Kao@ZIF, was washed with distilled water and dried in a Petri dish. To accelerate drying, the dish was covered with perforated Parafilm and placed in a fume hood overnight. This loading procedure was repeated in subsequent cycles as needed. Figure 2 presents a schematic illustration of the nanocarrier synthesis procedure, showing the formation of ZIF-8 and Kao@ZIF from zinc nitrate dihydrate and 2-mIM as the primary precursors.

### 2.7. Drug Loading of Nanocarriers

First, 1 mg of Naproxen was dissolved in 200 μL of EtOH using a vortex mixer to prepare a 10% (*w*/*w*) drug solution. Then, 10 mg of ZIF or Kao@ZIF was dispersed in 5 mL of EtOH and stirred magnetically for 1 h. Following this, 200 μL of the Naproxen solution (containing 1 mg of drug) was added to the ZIF dispersion and stirred at 200 rpm for 2 h [11]. The mixture was transferred into 50 mL falcon tubes and centrifuged at 4000 rpm for 20 min. A sample of the supernatant was collected using a micropipette to measure the drug concentration. The resulting precipitate, referred to as Nap-ZIF, was washed with distilled water and placed in a Petri dish. The Petri dish was covered with perforated parafilm and left to dry overnight in the hood. The drug loading efficiency (DLE) was calculated using the concentration values obtained from the supernatant, according to Equation (3).

(2) $$\mathrm{Drug Loading Efficiency}\left(\%\right)=\frac{\mathrm{Amount of drug loaded}}{\mathrm{Initial amount of drug}}\times 100$$

The drug release profile and pH responsiveness of the Naproxen-loaded ZIF and Kao@ZIF composites embedded in the PLA-gelatin hydrogel were evaluated based on the protocols described by Sun et al. [5] and Cao et al. [15]. For drug release analysis, each gelatin hydrogel sample was immersed in 100 mL PBS (pH 7.4) in an orbital shaker at low speed. Aliquots were collected daily over a 15-day period, stored in vials, and analyzed using LC-M/MS to determine the cumulative drug release. For pH responsiveness, each hydrogel was placed in 100 mL of acetate buffer (pH 5.4) with identical shaking conditions. Daily samples were again collected for 15 days, stored, and analyzed similarly to measure Naproxen release under acidic conditions.

### 2.8. Characterization

The chemical composition of the prepared nanocarriers and hydrogel scaffolds was analyzed by attenuated total reflectance (ATR) Fourier-transform infrared spectroscopy (FT-IR) spectroscopy (ThermoScientific NICOLET iS10) (Waltham, MA, USA) with 64 scans in the range of at 3500–500 cm^−1^.

Scanning electron microscopy (SEM) analysis was performed using a Thermo Fisher Scientific Quanta650) (Waltham, MA, USA), after coating with a 50 nm thick gold layer and using ETD under vacuum. SEM images were captured at 20 kV with a 3.5 spot size. Energy-dispersive X-ray spectroscopy (EDS) was conducted using an Oxford Instruments INCAx-act detector (Oxford Instruments, Uedem, Germany) at an acceleration voltage of 15 kV. Prior to SEM and EDS analysis, all samples including Kao@ZIF, ZIF, PLA-hydrogel, Kao@ZIF loaded PLA-hydrogel and ZIF loaded PLA-hydrogel were carbon-coated using a POLARON CC7650 carbon coater (Quorum Technologies Ltd., Laughton, East Sussex, UK). Liquid Chromatography-Tandem Mass Spectrometry (LC-MS/MS; Agilent Technologies 6420 TripleQuad, Agilent Technologies Inc., Santa Clara, CA, USA) was used to measure both the amount of Naproxen loaded into the nanocarriers and the amount released during in vitro experiments.

To assess the swelling behavior and degradation rate of the fabricated PLA-gelatin hydrogels, samples were immersed separately in distilled water and PBS solution. Since the PLA scaffold was encapsulated within gelatin, the PLA weight was excluded from all calculations. Initial weights (Wo) were recorded after gently blotting excess surface moisture with a laboratory tissue. Samples were then immersed in 100 mL of solution and incubated at 37 °C. Changes in sample weight over time (W_t_) were monitored up to 120 min, until equilibrium was reached.

Depending on whether the sample swelled or degraded, the final weight either increased or decreased [16,17]. The swelling or degradation ratio at a specific time was calculated using Equation (3).

(3) $$\mathrm{Swelling or Degradation ratio}\left(\%\right)=\frac{Wo-Wt}{Wo}\times 100$$

The drug release mechanism was analyzed using the Ritger–Peppas model (Equation (4)) from the cumulative naproxen release data. The release fraction (M_t_/M_∞_) was normalized to unity, and log–log plots of the release fraction versus time were used to determine the release exponent (*n*) and kinetic constant (k) through linear regression. This model was employed to provide further insight into the release mechanism of ZIF and Kao@ZIF nanocarriers under different pH conditions.

(4) $$\frac{M_{t}}{M_{\infty }}=k\;t^{n}$$

### 2.9. In Vitro Cyctotoxicity Assay

These experiments were conducted to evaluate the cytotoxicity of various nanocarrier formulations and their corresponding hydrogels, which are essential parameters for assessing their potential in therapeutic applications. L929 mouse fibroblast cells were cultured in Dulbecco’s Modified Eagle Medium (DMEM) supplemented with 10% fetal bovine serum (FBS) and 1% penicillin–streptomycin–amphotericin B (PSA) as described by Karaca et al. [18]. The cells stored at −80 °C were thawed according to the manufacturer’s instructions and seeded into culture dishes. The cells were incubated at 37 °C in a 5% CO_2_ atmosphere, and the culture medium was refreshed every 48 h. When the cells reached approximately 80% confluency, they were detached using trypsin, centrifuged, and resuspended in fresh medium. The number of viable cells was determined using a hemotocytometer, and the cytotoxicity tests were conducted as triplicates (*n* = 3) in 96-well plates. The following samples were prepared as follows: free drug, ZIF, Kao@ZIF, ZIF–Naproxen, Kao@ZIF–Naproxen, Hydrogel–ZIF, Hydrogel–Kao@ZIF, Hydrogel–ZIF–Naproxen, and Hydrogel–Kao@ZIF–Naproxen. For the nanocarrier groups, 20 mg of each formulation was suspended in 1000 µL of DMEM. For the release of the drug molecules, the prepared solutions were incubated in an orbital shaker. Afterwards, 2 mL of each solution was collected into falcon tubes. From each, 200 µL was transferred into eppendorf tubes and diluted 1:4 with fresh DMEM. As controls, positive (cells + medium) and negative (medium only) groups were included in the 96-well plates. For the assay, 200 μL of each test sample was added to the first well of each row, followed by three consecutive 1:2 serial dilutions into the adjacent wells.

## 3. Results

### 3.1. Structural Characterization of ZIF and Kao@ZIF Nanocarriers via FT-IR Analysis

Zeolitic Imidazolate Frameworks (ZIFs), synthesized from zinc acetate dihydrate and 2-methylimidazole (2-mIM), along with natural clay materials such as kaolin, are promising candidates for use in biomedical nanocarrier systems, particularly for applications like wound dressings and localized drug delivery. When integrated with poly(lactic acid) (PLA), these materials contribute to scaffold architectures that enhance structural integrity, promote cell proliferation, and support tissue regeneration. The biodegradability and mechanical properties of PLA further complement long-term in vivo applications.

**Figure 3:**
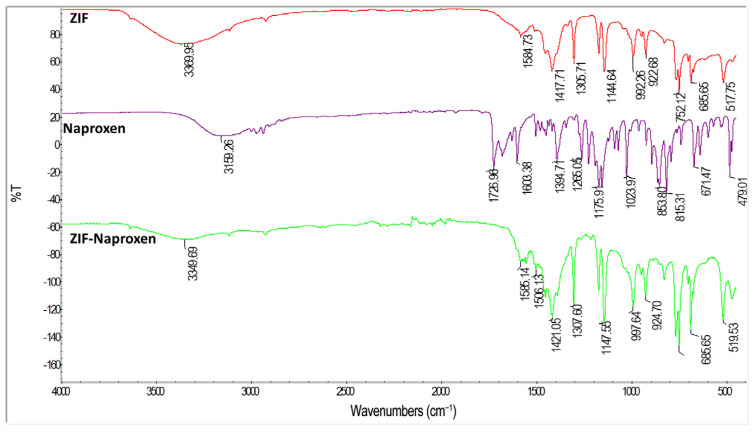
Comparative FT-IR spectra of pure ZIF, pure Naproxen, and ZIF-Naproxen composite confirming composite formation.

The successful synthesis of ZIFs was confirmed by FT-IR spectroscopy, which revealed characteristic peaks corresponding to -N-H stretching, C=C and C=N stretching, as well as -N-H bending vibrations (Figure 3). Similarly, the FT-IR spectrum of pure Naproxen exhibited characteristic bands such as -Ar-H bending, C-O bending, C=O stretching, and COOH vibrations (-Ar refers to aromatic groups), serving as critical references for identifying Naproxen in the drug-loaded nanocarrier systems (Supplementary Figure S1).

**Figure 4:**
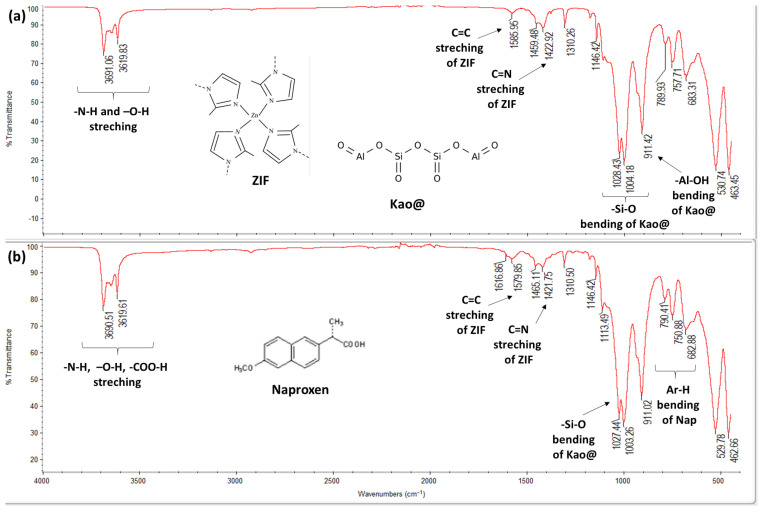
FT-IR spectra: (**a**) Kao@ZIF and (**b**) Kao@ZIF-Naproxen confirming Naproxen loading.

The Kao@ZIF composite spectrum (Figure 4), demonstrated peaks from both ZIF and kaolin components:

- ZIFspecific bands: NH and OH stretching, C=C and C=N stretching
- Kaolinspecific bands: SiO bending and AlOH bending

These distinct spectral features confirm the successful integration of ZIF structure onto the kaolin surface, validating the formation of the Kao@ZIF composite.

- In the FTIR spectrum of Kao@ZIFNaproxen, several characteristic bands corresponding to Naproxen (e.g., C=O stretching at ~1720 cm^−1^ and COOH vibration bands) were observed, which were absent in the pristine Kao@ZIF spectrum. This confirms the successful incorporation of Naproxen within the Kao@ZIF matrix. Additionally, slight shifts in ZIFrelated peaks (such as C=N and NH stretching vibrations) suggest possible interactions between the drug and the ZIF, likely through hydrogen bonding or coordination with zinc ions. These spectral changes further support the formation of a stable drug–carrier composite structure (Figure 4).

### 3.2. Morphological and Elemental Characterization (SEM-EDS)

**Figure 5:**
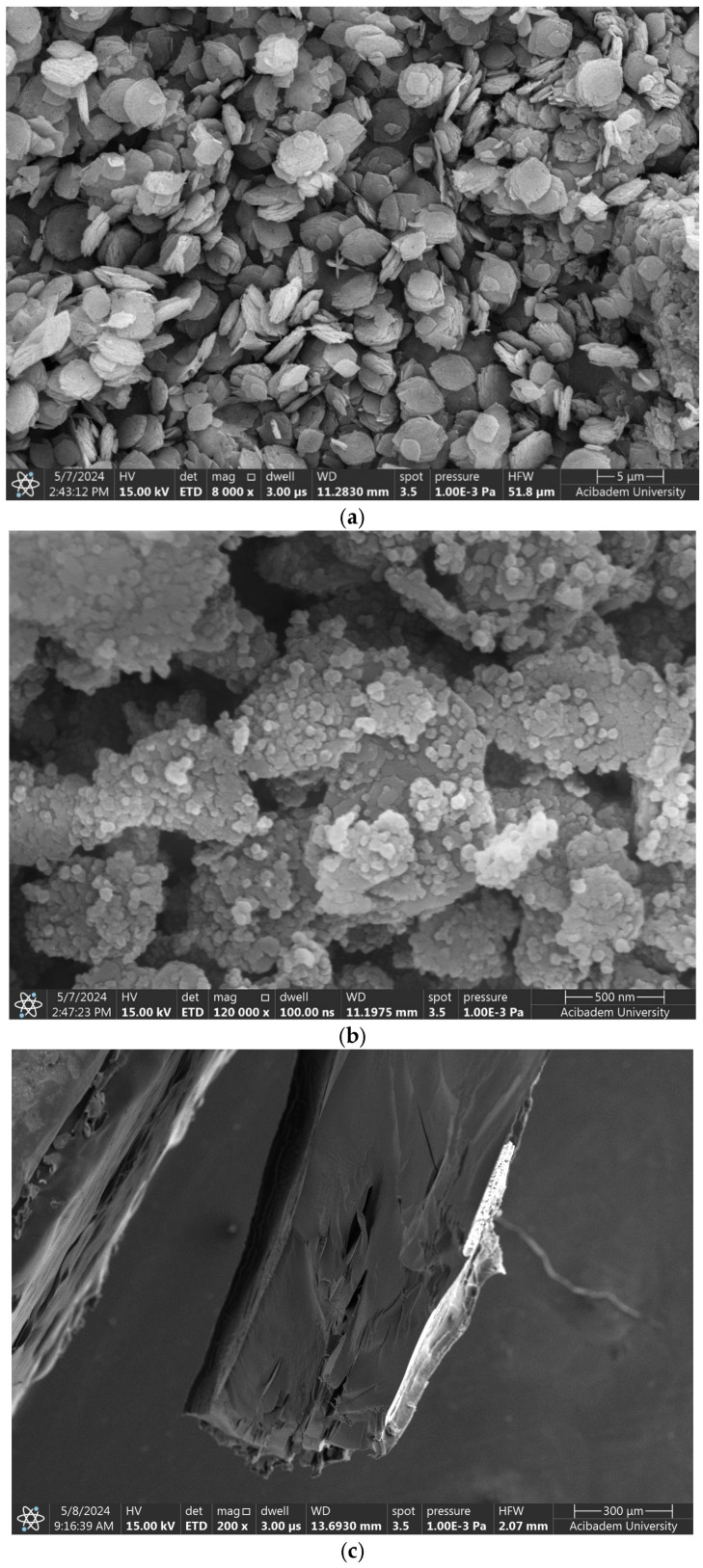
SEM images of (**a**) ZIF nanocarriers, (**b**) Kao@ZIF hybrids, and (**c**) PLA–gelatin interface.

To investigate the morphological and surface characteristics of the synthesized nanocarriers and their composite formulations, scanning electron microscopy (SEM) and energy-dispersive X-ray spectroscopy (EDS) analyses were conducted. The synthesized ZIF particles exhibited a sheet-like morphology (Figure 5a), with sizes ranging from 451.8 nm to 1.53 μm. This observation is consistent with previous findings [13] and is further supported by the particle size distribution shown in Supplementary Figure S2. Kao@ZIF nanocarriers, shown in Figure 5b, presented a relatively uniform and cubic-like morphology with particle sizes ranging from 48.6 nm to 55.5 nm. The SEM images confirmed the successful deposition of ZIF crystal on the kaolin surface, indicating the effective formation of a hybrid nanostructure. Compared to other clay-based supports like halloysite nanotubes reported in previous studies [14] the non-tubular layered structure of kaolin still enabled efficient crystal deposition, albeit with potential differences in surface area and morphology. Additionally, the morphology of a synthesized Kao@ZIF composite, prepared as a control nanocarrier system, is presented in Supplementary Figure S3. The SEM image reveals uniform ZIF deposition on the Kao@surface, demonstrating a successful hybrid coating approach.

**Figure 6:**
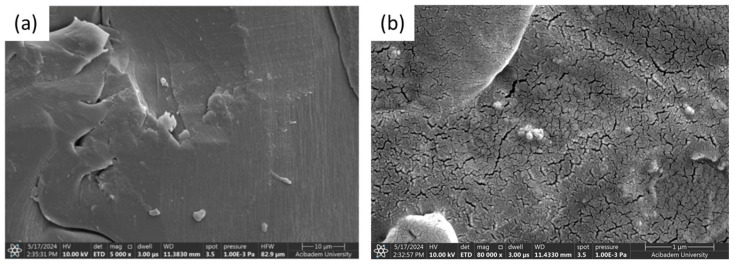
SEM Images comparing the surface morphology of (**a**) Naproxen-loaded ZIF/PLA–gelatin composite and (**b**) Naproxen-loaded Kao@ZIF/PLA–gelatin hydrogel.

The microstructure of the gelatin hydrogel revealed densely packed polymer layers and limited pore size, suggesting a high crosslinking density that enhances mechanical stability. The PLA–gelatin interface is illustrated in Figure 5c, with PLA appearing as the denser region on the left. Figure 6a,b show Naproxen-loaded ZIF and Kao@ZIF nanocarriers embedded in PLA–gelatin hydrogels, where nanocarrier aggregates are visible on the surface, indicating effective encapsulation and drug loading. Although the nanoparticles are partially embedded within the hydrogel matrix and may not appear as distinct entities at lower magnifications, the presence of characteristic granular surface features that are absent in the pure hydrogel strongly indicates successful incorporation of the nanocarriers into the scaffold.

#### Elemental Composition Was Determined by EDS Analysis

**Figure 7:**
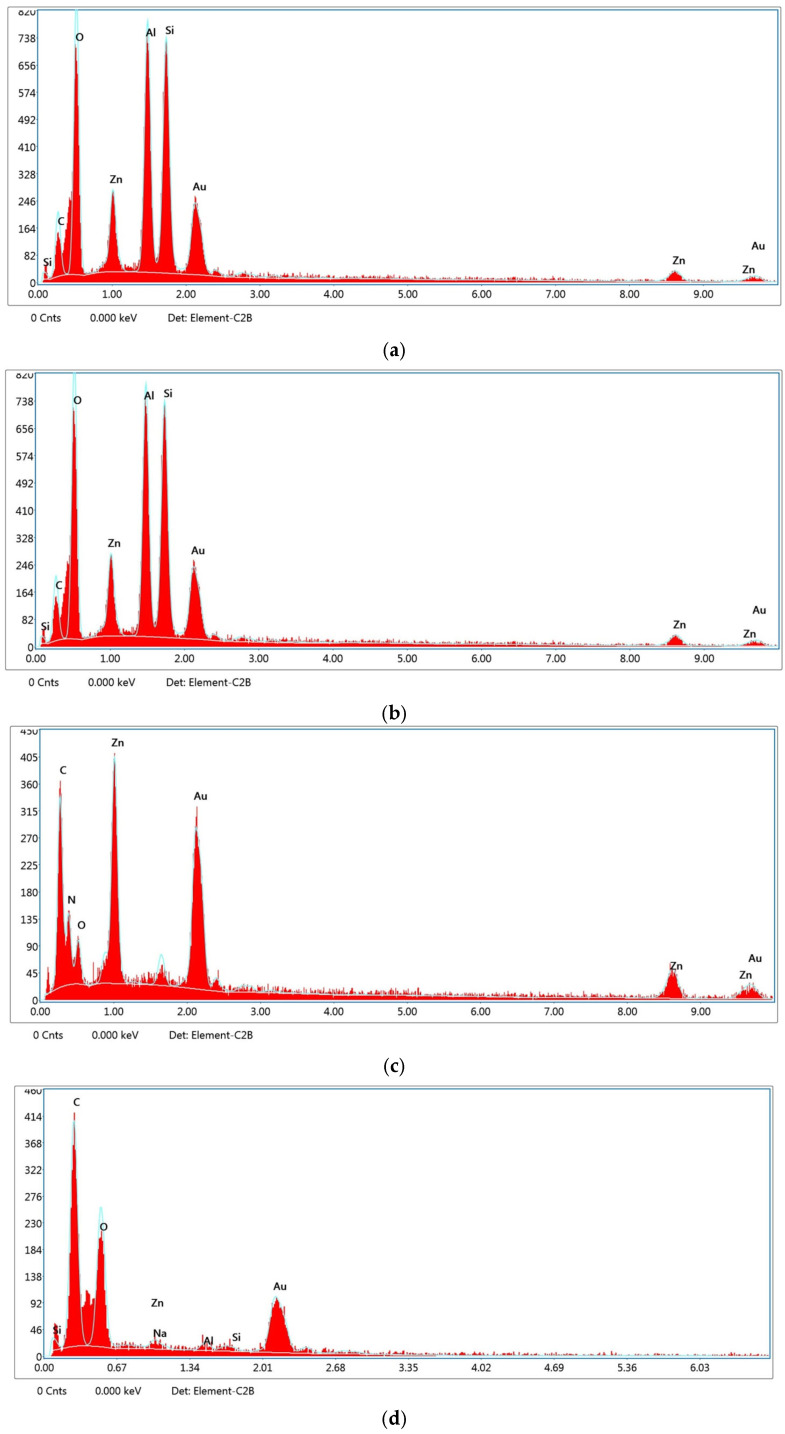
EDS spectra of (**a**) gelatin hydrogel, (**b**) Kao@ZIF nanocarriers, (**c**) ZIF-only nanocarriers, and (**d**) Kao@ZIF in gelatin hydrogel.

Energy-dispersive X-ray spectroscopy (EDS) analysis was conducted to verify the elemental composition and confirm the successful synthesis and integration of the nanocarrier systems into the PLA–gelatin hydrogel matrix. In the case of the gelatin hydrogel alone (Figure 7a), peaks corresponding primarily to carbon (C), oxygen (O), and gold (Au) were observed. The presence of Au is attributed to the gold sputter coating applied during SEM sample preparation, while C and O reflect the organic composition of gelatin. The EDS spectrum of the Kao@ZIF nanocarriers (Figure 7b) showed distinct peaks for aluminum (Al), silicon (Si), and zinc (Zn), confirming the successful combination of kaolin and ZIF components within the hybrid structure. For the ZIF-only samples (Figure 7c), a strong Zn signal was detected, consistent with the presence of zinc-based metal–organic framework structures. When Kao@ZIF nanocarriers were embedded in the gelatin hydrogel matrix (Figure 7d), the continued presence of Al, Si, and Zn signals confirmed the structural stability and uniform distribution of the nanocarriers within the composite. The EDS spectrum further confirmed the elemental composition of Naproxen-loaded ZIF was obtained from the EDS spectrum presented in Supplementary Figure S4, which revealed the continued presence of Zn peaks, indicating the structural integrity of the ZIF after drug loading. Overall, EDS data verified that the elemental fingerprints of both ZIF and kaolin were preserved in the composite systems, supporting the successful fabrication of the drug delivery platforms with consistent chemical integrity across all formulations.

EDS analysis further validates the successful fabrication of the Kao@ZIF composite by confirming the simultaneous presence of Zn (from ZIF) and Al/Si (from kaolin). This coexistence of characteristic elements provides direct evidence of the hybrid structure, complementing the morphological observations from SEM images.

### 3.3. Drug Loading Efficiency of ZIF and Kao@ZIF Systems

**Figure 8:**
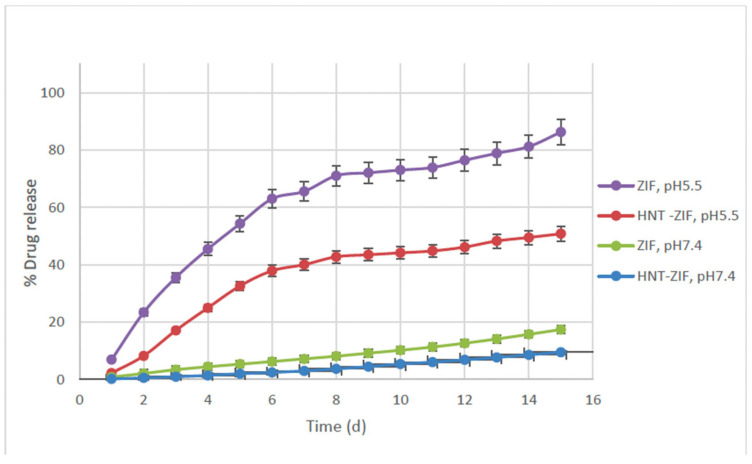
Cumulative drug release (%) of Naproxen from ZIF–Naproxen and Kao@ZIF–Naproxen systems embedded in PLA–gelatin hydrogel, tested in pH 7.4 and pH 5.5 buffers over 15 days.

Naproxen loading efficiencies exceeded 90% in both the ZIF and Kao@ZIF systems, highlighting their strong potential as nanocarriers for NSAIDs. Kao@ZIF consistently demonstrated slightly higher drug loading capacity compared to ZIF, likely due to its increased surface area and porous structure (Figure 8). This performance underscores the promise of Kao@ZIF as an effective platform for encapsulating poorly water-soluble drugs like Naproxen. In the first loading experiment, drug encapsulation efficiencies were calculated as 92.17% for ZIF–Naproxen and 92.95% for Kao@ZIF–Naproxen embedded into PLA–gelatin hydrogels. Similarly, free-form nanocarriers (not embedded in hydrogel) showed 91.48% efficiency for ZIF and 93.49% for Kao@ZIF. Table 1 summarizes drug loading efficiencies following the initial and secondary loading cycles.

In the second loading trial, ZIF-based hydrogels demonstrated a slight increase in efficiency (95.05%), whereas Kao@ZIF-based hydrogels showed a decreased value of 83.71%, potentially due to preparation inconsistencies or interfacial interference within the gel matrix (Table 1). Despite this variability, non-embedded drug-loaded carriers still exhibited excellent performance—96.59% for ZIF and 99.14% for Kao@ZIF, confirming the strong affinity of Naproxen to both frameworks. Integrating ZIF structures onto kaolin surfaces (forming Kao@ZIF hybrids) offers multiple advantages: it moderates burst release, enhances dispersion stability, and preserves the functional characteristics of the nanocarrier, all while aligning with pharmacopeial safety standards. These attributes position Kao@ZIF as a clinically relevant and structurally robust hybrid nanocarrier.

In this study, ZIF and Kao@ZIF nanocarriers were further incorporated into a PLA–gelatin hydrogel matrix, forming a pH-responsive biocomposite patch suitable for topical or wound-related drugs. While ZIFs contribute high drug loading efficiency and pH-sensitive release behavior, kaolin enhances mechanical integrity and offers regulatory familiarity, enabling a synergistic platform for sustained and localized delivery. Nonetheless, the inherently low adsorption capacity of kaolin may pose a limitation in applications requiring high drug payloads. Although kaolin remains favorable due to its structural integrity, widespread pharmaceutical acceptance, and regulatory compliance, its relatively modest drug retention performance indicates that the current system is still open to further optimization. One potential contributing factor is the limited incorporation of the active compound during the embedding process, which may reduce drug–carrier interactions or internal loading efficiency.

Despite these constraints, a notable strength of the Kao@ZIF system lies in its ability to maintain structural integrity throughout synthesis, drug loading, and incorporation into the PLA–gelatin matrix. The preservation of composite morphology and the absence of degradation during processing highlight the system’s mechanical and chemical robustness, an encouraging result for sustained drug delivery applications.

Future research should explore the refinement of this hybrid approach by leveraging clay minerals such as halloysite nanotubes (HNTs), which offer higher specific surface areas and a tubular structure that could further improve adsorption behavior and controlled release properties. Thus, while the Kao@ZIF-based system demonstrates promising characteristics, it remains a platform that is open to enhancement and evolution for broader therapeutic applicability.

### 3.4. pH-Responsive Drug Release Behavior

To assess the drug release profiles of the loaded drug and the pH-responsiveness of the nanocarriers, in vitro drug release studies were conducted over a 15-day period. Naproxen-loaded ZIF and Kao@ZIF nanocarriers embedded within PLA–gelatin hydrogels were incubated separately in two different buffer environments: phosphate-buffered saline (PBS, pH 7.4) and acetate buffer (pH 5.5), mimicking physiological and slightly acidic conditions, respectively.

Quantitative analysis was performed using LC-M/MS based on a calibration curve constructed using free Naproxen. The calibration curve for free Naproxen was constructed based on standard solutions, yielding the linear equation y = 57.8244x + 2217.7404 with a high correlation coefficient of R^2^ = 0.9808, indicating excellent linearity and reliability of the analytical method. A specific multiple reaction monitoring (MRM) analysis method was developed specific to Naproxen with an m/z value of 154.9 in the negative mode. The release profiles revealed that drug release from both nanocarrier systems was significantly higher under acidic conditions (pH 5.5) compared to neutral conditions (pH 7.4), with up to a 4.5-fold increase in cumulative release (Figure 8). These results confirm the pH-sensitive release behavior of both ZIF and Kao@ZIF systems, underscoring their potential for applications in acidic microenvironments such as inflamed or tumor tissues.

Between the two systems, Kao@ZIF exhibited slightly enhanced drug release rates under both pH conditions compared to ZIF alone. This effect can be attributed to the increased porosity and surface interaction provided by the kaolin matrix, which may facilitate more efficient drug diffusion. These findings suggest that integrating ZIF structures into kaolin substrates not only preserves the pH-responsive characteristics of the MOF but also contributes to a more sustained and controlled release pattern. In addition to the release data, it was observed that the PLA–gelatin hydrogel matrices preserved their physical integrity under both pH 5.4 and pH 7.4 conditions during the experimental period. No visible disintegration or collapse was detected, and the gels remained structurally stable while undergoing controlled swelling. This stability is consistent with the sustained and pH-sensitive release behaviors shown in Figure 8, confirming that the hydrogel matrices provide a reliable platform for drug delivery in different pH environments.

### 3.5. Kinetic Modeling

To further elucidate the release mechanism, the naproxen release profiles were fitted to the Ritger–Peppas model. The obtained kinetic parameters are summarized in Table 2. At pH 5.5, ZIF exhibited an *n* value of 0.83, indicating anomalous transport governed by a combination of diffusion and swelling. In contrast, Kao@ZIF presented an *n* value close to unity, suggesting Case II transport behavior, consistent with its more sustained and matrix-controlled release. At pH 7.4, both carriers showed *n* values ≥1.0, characteristic of Case II or super Case II transport, reflecting slower and more relaxation-controlled release mechanisms under physiological conditions. These results align well with the experimental release profiles and further confirm the stabilizing effect of kaolin incorporation into the nanocarrier system.

### 3.6. Swelling and Degradation Behavior of PLA-Gelatin Hydrogels

**Figure 9:**
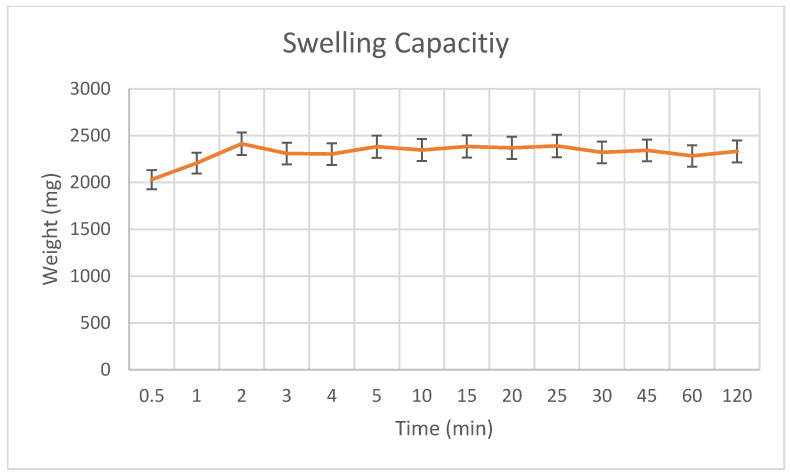
Swelling capacity of PLA-gelatin hydrogel.

To evaluate the fluid absorption capacity and potential release kinetics of the hydrogel matrix, the swelling behavior of the PLA–gelatin composite was assessed in vitro. The hydrogel exhibited rapid fluid uptake, reaching a peak swelling capacity of 2822.1 mg within the first 2 min (Figure 9). After this point, the swelling plateaued and remained stable for at least 2 h, with no remarkable mass loss or degradation. This rapid fluid absorption followed by structural equilibrium indicates that the PLA–gelatin matrix possesses excellent integrity and retention properties. Notably, this early swelling phase is critical for topical and localized drug delivery applications, where the hydrogel must quickly absorb exudates or surrounding fluids while maintaining mechanical stability and enabling a controlled release profile during the initial therapeutic window [19].

The average dry weights of the PLA-only scaffolds, PLA–gelatin coated scaffolds, and PLA–gelatin hydrogels are summarized in Supplementary Table S1, highlighting the increase in mass upon gelatin and hydrogel integration. The weight gain observed across samples reflects successful hydrogel incorporation and further supports the composite’s swelling capacity. It is important to note that the swelling behavior of the PLA–gelatin hydrogel does not directly affect the drug loading efficiency, since naproxen was loaded into the ZIF and Kao@ZIF nanocarriers prior to embedding. However, hydrogel swelling strongly influences the release stage: by absorbing fluid and expanding its network, the hydrogel increases porosity and creates diffusion channels that facilitate the controlled release of the drug from the nanocarriers. This highlights that swelling is a critical factor for regulating drug release rather than initial loading.

### 3.7. Cytotoxicity Assay

**Figure 10:**
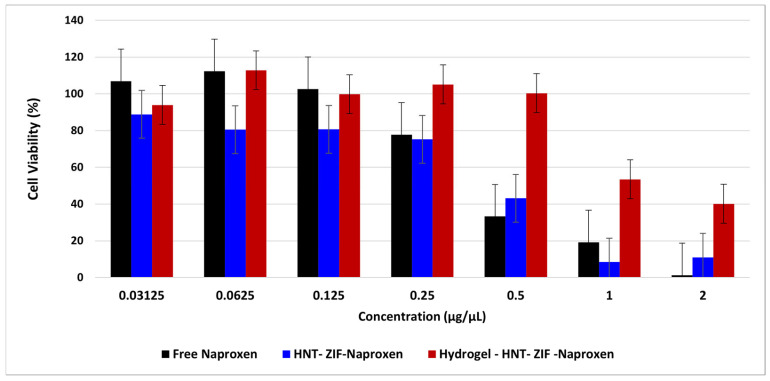
Cell viability results of free naproxen and its hydrogel loaded samples. Results are presented as mean ± SD (*n* = 3).

The CCK-8 cell viability assay was performed against L929 mouse fibroblast cell line for evaluating the cytotoxicity of prepared samples, together with their control groups. Drug concentrations ranging from 2 to 0.031 μg/mL were tested on fibroblasts for 24 h incubation. Free Naproxen (NAP) was seen to give a dose-dependent cytotoxicity, especially for higher doses, which yielded 0.349 µg/mL as EC_50_ value. As carriers, ZIF and Kao@ZIF samples were in non-toxic regions, up to 5.5 and 1.4 μg/mL. On the other hand, their drug-loaded versions (ZIF-NAP and Kao@ZIF-NAP) were found to improve the cell viability by shifting the threshold concentration of drug to more non-toxic region. As illustrated in Figure 10, the ZIF-NAP sample still remains in the safe cytotoxicity range up to 5.5 μg/mL, while their Kao@-added versions seem to provide more cell viability up to 2.75 μg/mL. These results suggest the ameliorating effect of this hydrogel system with drug-bearing Kao@ and ZIF complex, indicating the controlled release of free drug molecules to the environment.

## 4. Discussion

Nanocarriers represent a significant development in drug delivery systems, providing improved therapeutic efficacy, elevated bioavailability, and the targeted distribution of pharmacological substances. Nanoscale vehicles, including liposomes, polymeric nanoparticles, and metal–organic frameworks (MOFs), are engineered to enhance pharmacokinetics and pharmacodynamics by controlled drug release, increased solubility, and less systemic toxicity. A key characteristic of nanocarriers is their capacity to precisely regulate medication release via customizable structural properties. Zhang et al. [20] reported that the release kinetics of Celastrol from polyethylene glycol (PEG) derivatives were markedly affected by the steric hindrance of the outer shell, highlighting the direct impact of nanocarrier architecture on drug release. Soomherun et al. [21] demonstrated that the porous characteristics of carboxymethyl cellulose and PLGA-based nanocarriers enabled the controlled release of Nicardipine hydrochloride in accordance with a first-order kinetic model, whereby the elevated polymer concentration enhanced porosity and affected drug diffusion rates. Biocompatibility and targeted delivery are critical components in the advancement of contemporary nanocarriers. Strategies like PEGylation—binding PEG chains to nanoparticle surfaces—prolong systemic circulation duration and diminish immunogenicity [22]. Moreover, erythrocyte membrane-camouflaged nanocarriers have demonstrated enhanced tumor infiltration and cellular absorption, which is essential for cancer treatment [23].

Zeolitic Imidazolate Framework-8 (ZIF-8), a subtype of metal–organic frameworks (MOFs), has garnered considerable interest among the developing nanocarrier platforms. ZIF-8 exhibits significant porosity, superior biocompatibility, and inherent pH sensitivity, rendering it especially appropriates for regulated drug release in pathological conditions like tumors or inflammatory areas [24]. The pH-responsive behavior arises from the protonation of ZIF-8’s imidazolate ligands under acidic circumstances, leading to framework disintegration and subsequent drug release. The incorporation of amine groups has been documented to enhance the stability and compatibility of ZIF-8, hence facilitating improved drug loading and retention [25].

Lipid-based nanocarriers demonstrate potential, especially in traversing biological barriers such as the blood–brain barrier. Feng et al. [26] and Zhao et al. [27] demonstrate that these systems enhance drug solubility and targeting efficacy, whereas ligand surface changes promote receptor-mediated uptake by Priya et al. [28]. Besides independent nanocarriers, composite systems have also garnered attention. The use of kaolin, a naturally occurring biocompatible clay, with ZIF-8 presents synergistic benefits. The layered silicate structure of kaolin improves mechanical stability and offers an additional matrix for prolonged drug release [27]. The composite formed with ZIF-8 leverages ZIF-8’s substantial drug-loading capacity and pH-responsive release characteristics, while kaolin contributes to reducing premature drug leakage and extending systemic retention.

The ZIF-8/kaolin hybrid has been emphasized as a versatile platform for biomedical applications. It has been suggested that these composites can efficiently navigate physiological obstacles within the gastrointestinal tract and demonstrate tissue-specific targeting, hence improving therapeutic efficacy and minimizing side effects [29]. The hybrid system enhances the mechanical durability of the carrier and modifies surface properties critical for efficient drug interaction and biodistribution. Furthermore, the integration of stimuli-responsive mechanisms (such as pH- and temperature-sensitive release systems) enhances medication delivery. These intelligent carriers can initiate payload release under tumor-specific conditions (acidic pH or hyperthermia), offering spatial and temporal regulation of therapeutic activity [30,31]. The integration of kaolin and ZIF-8 into a cohesive nanocarrier platform corresponds with contemporary advances in precision medicine. This hybrid methodology utilizes the structural adaptability and responsiveness of metal–organic frameworks (MOFs) alongside the stability and biocompatibility of natural clays, signifying a promising strategy for advanced drug delivery systems.

## 5. Conclusions

This study successfully synthesized and characterized ZIF and Kao@ZIF nanocarriers, and integrated them into a PLA–gelatin hydrogel matrix, forming a novel pH-responsive drug delivery platform. It had a high loading capacity and strong structural integrity, highlighting its suitability for localized NSAID administration. The developed systems exhibited excellent drug loading efficiencies (>90%), pH-responsive release profiles, and structural robustness, especially in composite forms. The Kao@ZIF hybrid system showed additional advantages by combining ZIF’s efficient release characteristics with kaolin’s mechanical reinforcement and regulatory familiarity. These findings support the potential of ZIF-based composite carriers as viable platforms for localized and sustained delivery of poorly soluble drugs such as Naproxen.

## Figures and Tables

**Table 1:** Drug loading efficiency after initial and secondary loading cycles.

Samples	1st Loading Efficieny (%)	2nd Loading Efficieny (%)
ZIF- Naproxen in PLA—Hydrogel	92.17	95.50
Kao@ZIF- Naproxen in PLA—Hydrogel	92.95	83.71
Naproxen—ZIF (free-form)	91.48	96.59
Naproxen—Kao@ZIF (free-form)	93.49	99.14

**Table 2:** Ritger–Peppas kinetic parameters (*n* and k) for naproxen release from ZIF and Kao@ZIF nanocarriers at different pH values.

System	pH	*n*-Value	k-Value
ZIF	5.5	0.83	0.35
Kao@ZIF (HNT–ZIF)	5.5	1.00	0.22
ZIF	7.4	1.04	0.06
Kao@ZIF (HNT–ZIF)	7.4	1.11	0.04

*n*: release exponent; k: kinetic constant.

## Data Availability

All relevant data are included in the article and its Supplementary Materials. Additional data are available from the corresponding author upon reasonable request.

## Supplementary Materials

The following supporting information can be downloaded at: https://www.mdpi.com/article/10.3390/polym17182497/s1, Figure S1: FT-IR spectra of synthesized ZIF (a) and pure Naproxen (b), highlighting characteristic peaks associated with each material; Figure S2: Particle size distribution of the synthesized ZIF, confirming nanoscale particle formation suitable for biomedical applications. Figure S3. SEM image of synthesized HNT-ZIF composite showing uniform ZIF deposition on the HNT surface and approximate particle dimensions; Figure S4. EDS spectrum of Naproxen-loaded ZIF embedded in PLA–gelatin hydrogel, showing preserved Zn signal and confirming the structural integrity of ZIF after drug incorporation; Figure S5. in vitro Cytotoxicity experiment results for Hydrogel-ZIF (A), ZIF-Naproxen (B) and Hydrogel-ZIF-Naproxen (C), indicating the improved cell viability for drug molecules after their encapsulation into hydrogel scaffold; Table S1. Average dry weights of PLA-only scaffolds, PLA–gelatin coated scaffolds, and PLA–gelatin hydrogels, demonstrating increased mass following gelatin and hydrogel integration.

## Abbreviations

The following abbreviations are used in this manuscript:

ZIF	Zeolitic imidazolate frameworks
Kao@	Kaolin
Kao@ZIF	Kaolin-ZIF
PLA	Polylactic acid
MOF	Metal–organic frameworks
NSAID	Nonsteroidal anti-inflammatory drug
ATR	Attenuated total reflectance
FT-IR	Fourier-transform infrared
SEM	Scanning electron microscopy
LC-M/MS	Liquid Chromatography-Tandem Mass Spectrometry
DMEM	Dulbecco’s Modified Eagle Medium
FBS	Fetal bovine serum
PSA	Penicillin–streptomycin–amphotericin
EDS	Energy-dispersive X-ray spectroscopy
EDL	Drug loading efficiency
