# Supplementary material for: pH-Sensitive Naproxen Delivery via ZIF and Kaolin@ZIF Nanocarriers in 3D-Printed PLA–Gelatin Hydrogels
Source: Polymers (Basel). 2025 Sep 16;17(18):2497. doi: 10.3390/polym17182497 (PMC12473257; doi:10.3390/polym17182497)
Supplement: Supplementary file 1 [file polymers-17-02497-s001.zip › polymers-3824907-supplementary.pdf]

Supplementary Files

Supplementary Figure S1. FT-IR spectra of the synthesized ZIF (a) and pure Naproxen (b), highlighting the characteristic peaks associated with each material.

Supplementary Figure S2. Particle size distribution of the synthesized ZIF, confirming nanoscale particle formation suitable for biomedical applications.

Supplementary Figure S3. SEM image of the synthesized HNT–ZIF composite showing uniform ZIF deposition on the HNT surface and approximate particle dimensions.

Supplementary Figure S4. EDS spectrum of Naproxen-loaded ZIF embedded within the PLA–gelatin hydrogel, showing preserved Zn signals and confirming the structural integrity of ZIF after drug incorporation.

Supplementary Figure S5. *In vitro* cytotoxicity experiment results for Hydrogel–ZIF (A), ZIF-Naproxen (B), and Hydrogel–ZIF–Naproxen (C), indicating improved cell viability with drug molecules after encapsulation into the hydrogel scaffold.

Supplementary Table S1. Average dry weights of PLA-only scaffolds, PLA–gelatin coated scaffolds, and PLA–gelatin hydrogels, demonstrating increased mass following gelatin and hydrogel integration.

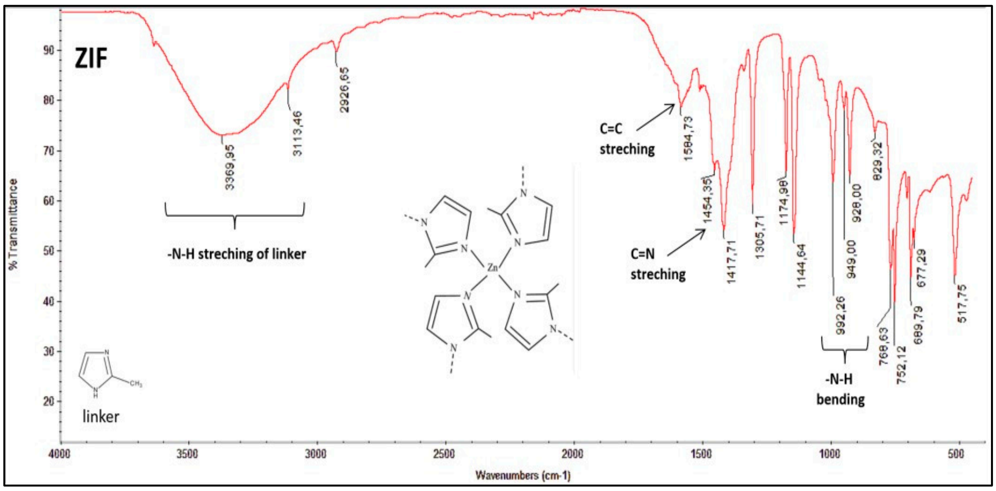

(a)

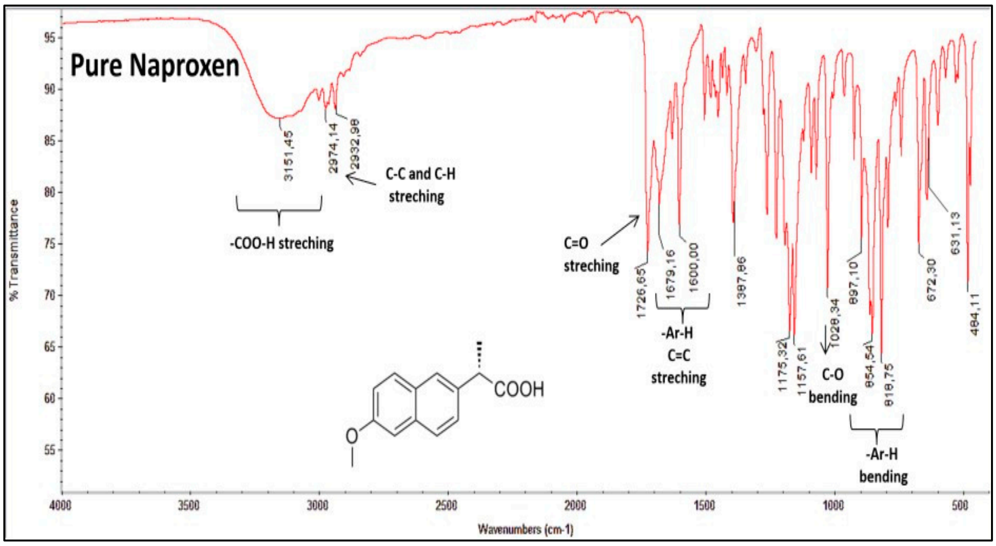

(b)

Supplementary Figure S1. FT-IR spectra of synthesized ZIF (a) and pure Naproxen (b), highlighting the characteristic peaks associated with each material.

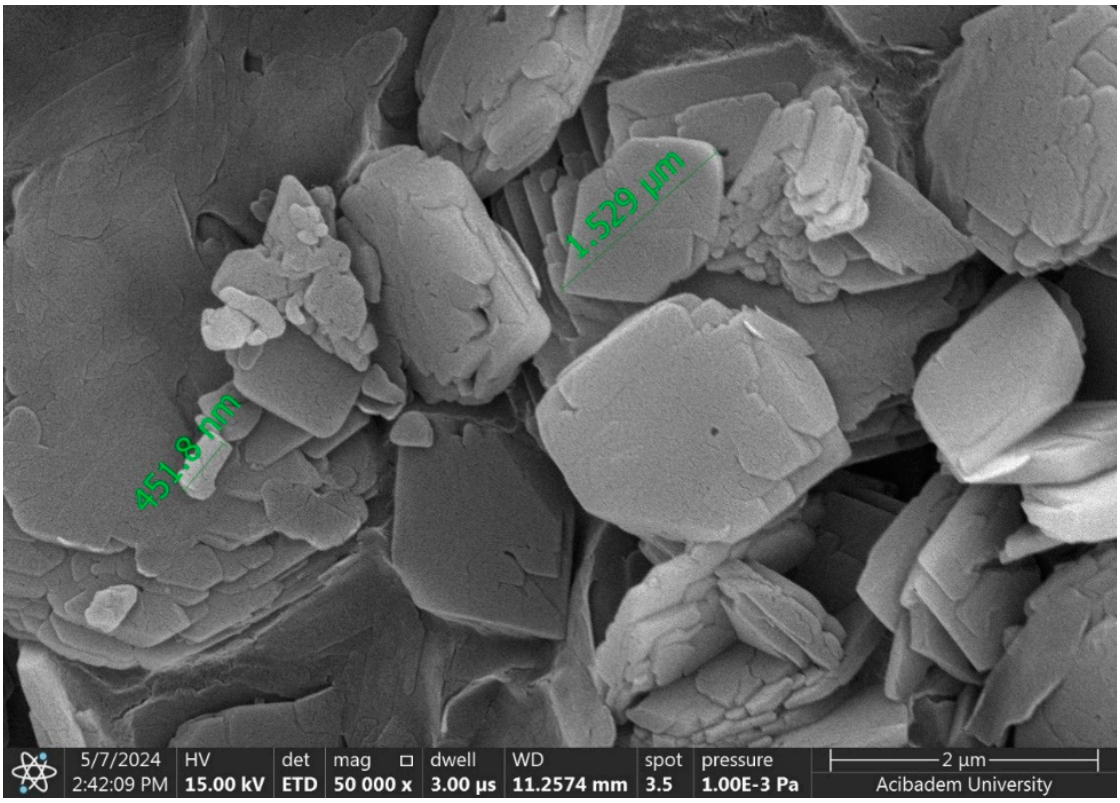

Supplementary Figure S2. Particle size distribution of the synthesized ZIF, confirming nanoscale particle formation suitable for biomedical applications.

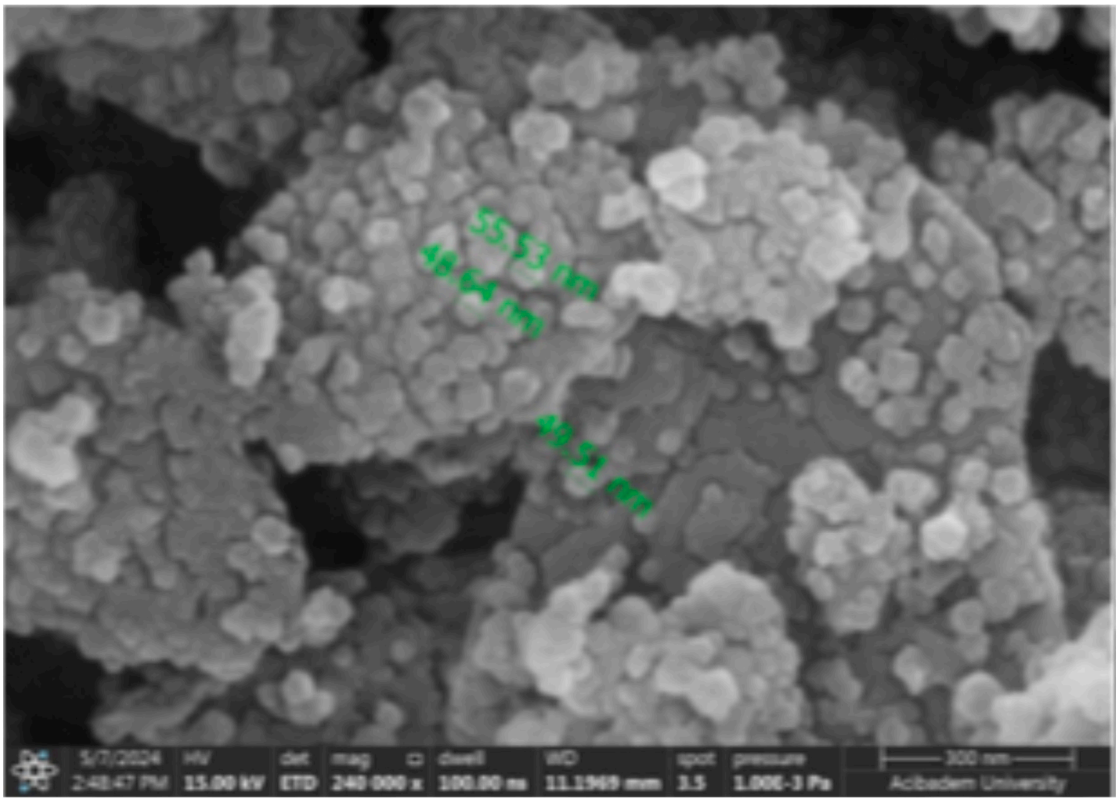

Supplementary Figure S3. SEM image of the synthesized HNT–ZIF composites showing uniform ZIF deposition on the HNT surface and approximate particle dimensions.

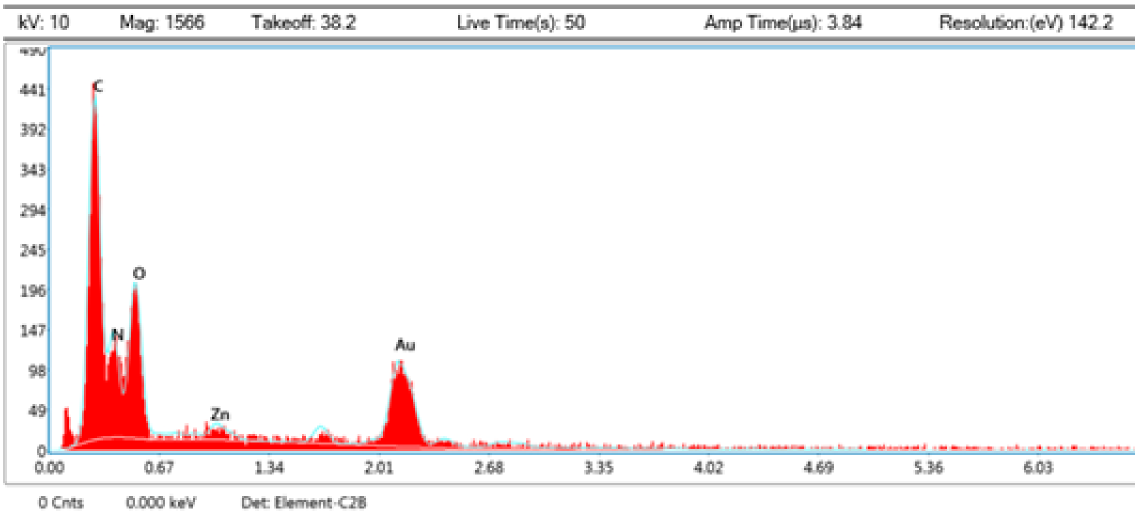

Supplementary Figure S4. EDS spectrum of Naproxen-loaded ZIF embedded within the PLA–gelatin hydrogel, showing preserved Zn signals and confirming the structural integrity of ZIF after drug incorporation.

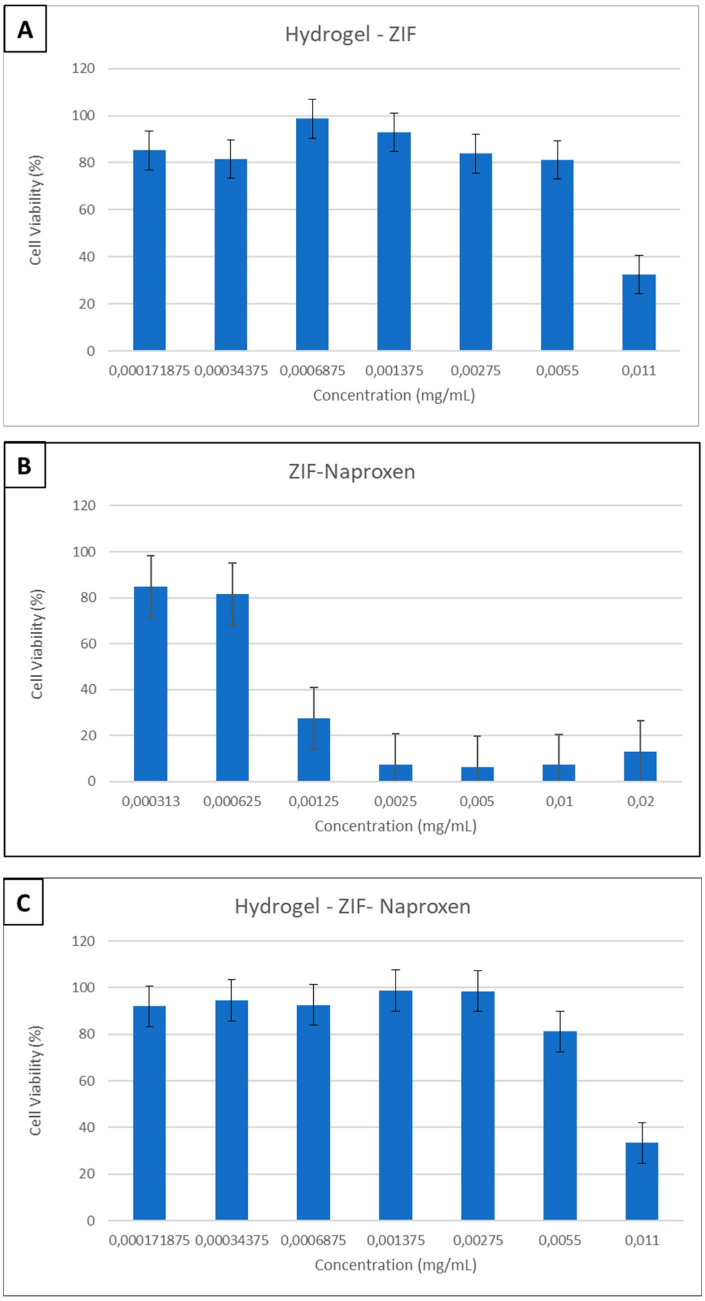

Supplementary Figure S5. *In vitro* cytotoxicity experiment results for Hydrogel–ZIF (A), ZIF–Naproxen (B), and Hydrogel–ZIF–Naproxen (C), indicating the improved cell viability with drug molecules after encapsulation into the hydrogel scaffold.

Supplementary Table S1. Average dry weights of PLA-only scaffolds, PLA–gelatin-coated scaffolds, and PLA–gelatin hydrogels, demonstrating increased mass following gelatin and hydrogel integration.

Number of Samples	Dried PLA (mg)	Dried PLA–Gelatin (mg)	Dried PLA–Gelatin Hydrogel (mg)
1	893.5	1624.7	-
2	892.1	1874.4	1285.5
3	1042.4	1259.1	1733.1
4	848.1	1594.2	1297.5
5	828.9	1350.1	1383.1
6	792.9	1216.2	1277.6
7	865.4	1550.3	1550.3
8	925.4	1374.8	1374.8
9	738.1	1376.6	1376.6
10	785.7	1934.8	1413.9
11	1018.2	1934.8	1934.8
12	823.0	1540.0	1540.0
13	967.7	1571.7	1571.7
